# Current knowledge about immunotherapy resistance for melanoma and potential predictive and prognostic biomarkers

**DOI:** 10.20517/cdr.2023.150

**Published:** 2024-05-13

**Authors:** Lanni Song, Yixin Yang, Xuechen Tian

**Affiliations:** ^1^Wenzhou Municipal Key Laboratory for Applied Biomedical and Bio-pharmaceutical Informatics, Wenzhou-Kean University, Wenzhou 325060, Zhejiang, China.; ^2^Zhejiang Bioinformatics International Science and Technology Cooperation Center, Wenzhou-Kean University, Wenzhou 325060, Zhejiang, China.; ^3^College of Science, Mathematics and Technology, Wenzhou-Kean University, Wenzhou 325060, Zhejiang, China.; ^4^Dorothy and George Hennings College of Science, Mathematics and Technology, Kean University, Union, NJ 07083, USA.

**Keywords:** Melanoma, immunotherapy, resistance mechanism, tumor microenvironment, biomarkers

## Abstract

Melanoma still reaches thousands of new diagnoses per year, and its aggressiveness makes recovery challenging, especially for those with stage III/IV unresectable melanoma. Immunotherapy, emerging as a beacon of hope, stands at the forefront of treatments for advanced melanoma. This review delves into the various immunotherapeutic strategies, prominently featuring cytokine immunotherapy, adoptive cell therapy, immune checkpoint inhibitors, and vaccinations. Among these, immune checkpoint inhibitors, notably anti-programmed cell death-1 (PD-1) and anti-cytotoxic T lymphocyte antigen-4 (CTLA-4) antibodies, emerge as the leading strategy. However, a significant subset of melanoma patients remains unresponsive to these inhibitors, underscoring the need for potent biomarkers. Efficient biomarkers have the potential to revolutionize the therapeutic landscape by facilitating the design of personalized treatments for patients with melanoma. This comprehensive review highlights the latest advancements in melanoma immunotherapy and potential biomarkers at the epicenter of recent research endeavors.

## INTRODUCTION

Melanoma, recognized as an aggressive form of skin cancer, represents a substantial challenge to both patients and clinicians due to its propensity for relapse and metastasis^[1]^. In 2024, it is anticipated that there will be 100,640 new cases of melanoma in the United States, with an estimated 8,290 deaths^[2]^. In contrast, melanoma in China is relatively rare, leading to delayed diagnoses and treatments. Statistical reports from 2022 indicate approximately 8,800 new cases and 5,400 deaths in China, accounting for 61% of the cases^[3]^. Meantime, melanoma has emerged as the fifth most prevalent cancer in the European Union (EU) since 2020, representing 4% of new cancer cases and 1.3% of all cancer deaths. Notably, the mortality rate in men exceeds that in women^[4]^. The factor that is considered to be the most common cause of melanoma cancer is thought to be ultraviolet rays (UV)^[5]^. UV exposure causes DNA mutations in protective melanocytes^[6]^. Conventional chemotherapy has historically offered limited success, with a response rate of merely 10% and brief survival periods^[7]^. However, a groundbreaking shift occurred with the advent of immune checkpoint inhibitors (ICIs), which have revolutionized the prognosis for melanoma and various skin cancers. Immunotherapy has emerged as the standard adjuvant therapy for patients with high-risk resected stage III or IV melanoma, and its promising results extend to diverse skin cancer types^[8,9]^. The objective response rate (ORR) for immunotherapy treatment for melanoma has increased from 16% during the period of high-dose interleukin (IL)-2 therapy to 61% with the combination treatment of anti-programmed cell death-1 (PD-1) antibody Nivolumab and anti-cytotoxic T lymphocyte antigen-4 (CTLA-4) antibody Ipilimumab^[10,11]^. This improvement is attributed to the complex interplay of immune escape mechanisms, driven by the heterogeneity of melanoma cells both within and across tumors^[12,13]^. Furthermore, the genetic mutations resulting from this cellular heterogeneity may have clinical significance as promising biomarkers. This is because such specific mutations reflect the tumor’s characteristics of immune escape and response to treatment, thus providing a basis for personalized therapeutic strategies. Consequently, the urgent need to identify specific biomarkers that predict immunotherapy outcomes in melanoma and skin cancer patients has spurred intensive research in this field^[14]^. This review aims to provide a comprehensive overview of immunotherapy for melanoma, exploring its applications, benefits, mechanisms of resistance, and associated molecular biomarkers. We highlight the latest developments and future perspectives in melanoma research, aiming to offer insights into the evolving landscape of treatment strategies.

## STRATEGIES AND ADVANCES OF IMMUNOTHERAPY IN MELANOMA

Immunotherapy, a revolutionary treatment approach that harnesses the body’s immune system to combat cancer, has emerged as a transformative breakthrough in the management of skin cancers, particularly melanoma. Unlike traditional treatments like chemotherapy, which directly attack cancer cells, immunotherapy works by unleashing the power of the immune system to recognize and eliminate cancerous cells^[15,16]^. This innovative approach encompasses various strategies, such as immune checkpoint inhibitors, cytokine immunotherapy, adoptive cell therapy, and vaccinations. These techniques have demonstrated remarkable success in melanoma, with immune checkpoint inhibitors like anti-PD-1 and anti-CTLA-4 antibodies standing out due to their efficacy. Furthermore, adoptive cell therapy and tumor vaccinations also promise to expand treatment options. Overall, these advancements in immunotherapy offer new hope for patients who previously faced limited treatment options and grim prognoses.

### ICIs

ICIs represent a significant milestone in melanoma treatment, substantially improving the survival rates of patients. In 1996, the concept of immune checkpoint blocking was first introduced. Currently, anti-PD-1 antibodies and anti-CTLA-4 antibodies are among the most widely used immune checkpoint inhibitors, providing significant therapeutic benefits from either monotherapy or combination^[17-19]^.

PD-1/programmed cell death ligand 1 (PD-L1) interaction involves a ligand and receptor between tumor cells and T cells. The ligands PD-L1/PD-L2 on the surface of tumor cells specifically bind to the PD-1 receptor on the surface of T cells. This binding inhibits the immune recognition of tumor cells by T cells and causes immune escape^[20,21]^. Nivolumab and Pembrolizumab are FDA-approved anti-PD-1 monoclonal antibodies (mAb) used to treat melanoma. In July 2014, Nivolumab was approved in Japan for treating advanced melanoma, becoming the world’s first PD-1 inhibitor to receive regulatory approval. Pembrolizumab, developed by Merck Sharp & Dohme for treating unresectable or metastatic melanoma following ipilimumab therapy, effectively boosts the body’s immune response. They have been widely adopted in clinical practice, and there is a growing trend toward combining them with multiple drugs to enhance drug sensitivity or act as adjuvant or neoadjuvant therapy^[22,23]^. The results of a clinical trial using Relatlimab (LAG-3 inhibitor) and Nivolumab for neoadjuvant therapy in thirty patients with stage III resectable melanoma or single-metastatic stage IV melanoma showed that 57% of patients had complete remission and 70% had a partial response. This combination was well-tolerated during neoadjuvant therapy, although some toxicity was observed during adjuvant therapy^[24]^.

A retrospective study of blood sample analysis in patients with stage IIIb-IV melanoma after three months of combined therapy with cetirizine and anti-PD-1 antibodies showed that combining anti-PD-1 mAb with cetirizine can significantly improve the progression-free survival (PFS) (28 *vs.* 15 months; 30 *vs.* 15 months in ICI-treated patients) and overall survival (OS) (36 *vs.* 23 months; 40 *vs.* 22 months in ICI-treated patients) of patients who had received other ICI treatments^[25]^. Anti-PD-1 antibodies have also been used to treat different cancers, such as non-small cell lung cancer. Research demonstrated through meta-analysis that chemotherapy combined with anti-PD-1 antibody was superior to combination therapy with anti-PD-L1 antibody, with better OS (*P* = 0.022) and PFS (*P* = 0.029)^[26]^.

CTLA-4 is a transmembrane receptor on T cells, which modulates the immune response of T cells by binding to antigen-presenting cell surface receptors. Blocking CTLA-4 mAb can lead to a surge in T cell proliferation and their subsequent attack on tumor cells^[27,28]^. In 2011, the FDA approved Ipilimumab to treat unresectable advanced melanoma, with 5-year survival rates for naïve advanced melanoma patients treated with the ipilimumab monotherapy reported between 21.4% and 49.5%^[29]^. As adjuvant therapy for stage III melanoma after resection, ipilimumab has shown a 5-year recurrence-free survival rate of 40.8% and an OS of 65.4% at five years^[30-32]^.

In 2023, the American Society of Clinical Oncology (ASCO) issued an update to the guidelines for the systemic treatment of melanoma patients, introducing new recommendations for neoadjuvant pembrolizumab for patients with resectable stage IIIB-IV cutaneous melanoma, adjuvant nivolumab or pembrolizumab for stage IIB-C disease, and adjuvant nivolumab plus ipilimab as a potential option for stage IV disease. For patients with unresectable or metastatic cutaneous melanoma, Nivolumab + Relatlimumab (LAG-3 inhibitor ) was added as a potential option, regardless of BRAF mutation status, and nivolumab + ipilimumab plus nivolumab was highlighted as superior to BRAF/MEK inhibitor therapy in the first-line treatment of patients with BRAF-mutated disease^[33]^.

### Cytokine immunotherapy

Cytokine immunotherapy, rooted in the principle of modulating the body’s innate immune responses against malignancies, has been pivotal in reshaping the treatment landscape for melanoma. Among the array of treatments, interferons (IFNs) and high-dose IL-2 have traditionally been at the forefront of melanoma immunotherapy^[34,35]^. Specifically, high-dose IL-2 and interferon-alpha have been approved by the FDA as immunotherapy drugs for melanoma^[36]^. In addition, cytokines such as IL-10, IL-12, IL-15, IL-18, and IL-21 have been proven to mobilize immune regulation in treating other cancers^[37,38]^. These cytokines are often described as hormones, which play dual roles in regulating the immune system’s response against tumor cells and directly inducing cancer cell apoptosis, cell cycle arrest, and angiogenesis inhibition^[39]^. For instance, IFN-alpha is known to enhance the cytotoxicity and the antitumor ability of NK cells and strengthen CD8+ cytotoxic T lymphocytes (CTLs)^[40]^. Interferons have been used as adjuvant therapy for melanoma for many years, and the first clinical trial of IFN-2b as an adjuvant therapy that significantly improves high-risk melanoma OS and recurrence-free survival increased disease-free survival to 1.7 years and OS to 3.8 years^[41]^. Notably, IFN-alpha-1b, the first genetically engineered recombinant drug approved by the China Food and Drug Administration, has been proven effective in prolonging the survival of melanoma patients^[42]^.

In East Asia, where melanoma constitutes a smaller percentage of cancer cases and often goes undetected until advanced stages, the response rate to immune checkpoint inhibitors is notably lower. Combining ICIs with IFN has been explored to enhance response rates and therapeutic effects. A retrospective study evaluating the combination of IFN-alpha-1b and anti-PD-1 mAB in untreated stage IV melanoma patients observed a 32.8% objective response rate and 18-month overall survival. While the combination therapy was associated with a high rate of adverse events, less than 10% of patients experienced severe (grade ¾) adverse effects, significantly lower than those observed with the combination of ICI and IFN-alpha-2b^[43,44]^. The therapeutic landscape of melanoma is continuously evolving, with ongoing clinical trials and research focusing on harnessing the full potential of cytokine immunotherapy. The convergence of these therapies with other approaches, such as immune checkpoint inhibitors, offers hope for more tailored and effective treatment strategies for melanoma patients.

### Adoptive cell therapy

Adoptive cell therapy (ACT) represents a paradigm shift in cancer treatment, harnessing the patient’s immune cells for therapeutic purposes. This strategy offers the promise of a tailored approach, especially for patients who are refractory to traditional treatments. ACT is centered on manipulating a patient’s immune cells, amplifying their tumor-fighting capabilities, and then reintroducing them to the body to target and destroy cancer cells. The two primary types of ACT that have garnered attention in melanoma treatment are tumor-infiltrating lymphocyte (TIL) therapy and chimeric antigen receptor (CAR) T-cell therapy^[45-47]^. TIL therapy capitalizes on the presence of immune cells within the tumor microenvironment. By isolating these cells and stimulating their proliferation *ex vivo*, typically using agents like IL-2, clinicians can reinfuse a substantial number (around 10^11^) of these primed cells into the patient^[48]^. Historically, TIL’s debut in cancer treatment traces back to the 1980s, and its efficacy, as evidenced by an ORR of 41% in melanoma, attests to its potential^[49]^. On the other hand, CAR T-cell therapy involves genetic modification of T cells to express receptors specifically engineered to target tumor-associated antigens. This tailored recognition facilitates the precise targeting of cancer cells, paving the way for innovative therapeutic strategies.

To harness the full potential of TIL for personalized immunotherapy, ongoing research seeks to unearth a broader spectrum of antigens, encompassing tumor-associated, tumor-specific, and unconventional antigens, which include those antigens originate from non-coding regions of the genome or are produced through aberrant transcription, translation, or post-translational modification processes, from coding regions, whether wild-type or mutated^[50]^. This is particularly pivotal for patients demonstrating resistance or treatment failure with ICIs^[51]^. Notably, melanoma patients with BRAF and NRAS mutations who have failed to respond optimally to agents like anti-PD-1 or anti-CTLA-4 antibodies have exhibited either partial response or long-stable disease (SD) after receiving TIL treatment^[52]^.

In February 2024, Lifileucel received accelerated approval from the U.S. FDA, becoming the first approved TIL therapy to treat solid tumors. Lifileucel’s definitive clinical trial (NCT02360579) is an open-label, multi-cohort, multi-center Phase 2 clinical trial in patients with advanced melanoma who have progressed on or after PD-1/PD-L1 inhibitors. Based on the trial results, Lifileucel demonstrated significant clinical benefit, with an ORR of 31.4% as assessed by an independent review committee (IRC) at a median follow-up of 27.6 months, including patients with complete response (CR) and partial response (PR), and the most prolonged duration of response was 55.8 months^[53]^. The latest clinical trial results confirmed its efficacy in non-small cell lung cancer. In the Phase 2 clinical trial in patients with metastatic non-small cell lung cancer, Lifileucel demonstrated an objective response rate of 21.4% in patients who had undergone prior immunotherapy and observed responses in some refractory tumor types, despite some manageable adverse events and individual patient deaths due to treatment-related adverse events^[54]^.

However, the promise of ACT comes with challenges. The intricate process of cell extraction, modification, expansion, and reinfusion is time-consuming and costly. Before patients receive TIL infusions, a brief non-myeloablative (NMA) lymphodepletion regimen, usually chemotherapy, IL-2, or systemic radiation therapy, is required. This is so that after receiving the TIL infusion, lymphocyte deletion stimulates the body to enhance the effect of TIL^[55]^. Though it is an essential process, chemotherapy and IL-2 therapy are the most toxic parts of the treatment process. However, TILs that can generate autoimmune stimulation signals are being developed so that patients no longer need to be treated with IL-2, thereby reducing adverse effects. As the realm of ACT continues to evolve, the horizon is replete with opportunities from refining cell preparation techniques to exploring combinations with other therapeutic modalities. The journey toward optimizing ACT for melanoma patients is well underway.

### Vaccination for melanoma

Cancer vaccines are an alternative immunotherapy option for patients who tolerate ICIs. Cancer vaccines come in different forms with varying antigen types and contents, including peptide vaccines, whole cell vaccines, dendritic cell vaccines, neoantigen vaccines, nucleic acid (DNA, RNA) vaccines, and protein vaccines, among others^[56-59]^. Their ability to elicit an immune response against tumors makes them a viable alternative, especially for patients who might be tolerant or refractory to ICIs. A recent clinical trial of an immune-modulatory vaccine activates IDO/PD-L1 specific T cells, targets cancer cells by IDO- and PD-L1-specific CD8+ T cells, and releases pro-inflammatory cytokines by CD4+ T cells. The strategy combined with anti-PD-1 antibody showed 62.7%-90.5% of ORR with 27.4%-60.8% of complete responses^[60]^.

With deepening the understanding of tumor biology and immunology, personalized vaccines are becoming a research hotspot. These vaccines are tailored to the patient’s tumor characteristics and genetic background to improve the targeting and effectiveness of the vaccine. In clinical trial NCT01970358, eight patients with surgically resected IIIB/C or IVM1a/b melanoma obtained persistent cancer control after receiving personal neoantigen vaccination, NeoVax, a peptide vaccine targeting up to 20 antigens per patient. The peptide vaccine was designed by comparing whole-exome sequencing of tumor cells and normal cells of individual patients to find mutant peptides that can bind to HLA-A and HLA-B. NeoVax-induced T cells can specifically target melanoma and exhibit memory phenotype^[61,62]^. Another clinical trial for the liposomal RNA (RNA-LPX) vaccine, Fixvac, which encodes four tumor-associated antigens (TAAs) - New York oesophageal squamous cell carcinoma 1 (NY-ESO-1), melanoma-associated antigen A3 (MAGE-A3), tyrosinase, and transmembrane phosphatase with tensin homology (TPTE), showed a great response when vaccinated alone or combined with checkpoint inhibitors for ICI-experienced patients. Fixvac increased the release of cytokines, such as IFN-α, IFN-γ, IL-6, IFN-inducible protein (IP)-10, and IL-12 p70 subunit in plasma at a relatively low concentration in humans compared to what was required to achieve such immune outcomes in mice^[57]^. Another neoantigen-based vaccine, NEO-PV-01, combined with or without anti-PD-1 antibody in an Ib clinical trial of 60 patients who received the vaccine, ORR was 59% (39%-78%), 39% (17%-64%), and 27% (8%-55%) in melanoma, non-small cell lung cancer (NSCLC), and bladder cancer patients, respectively. No significant adverse effects were found^[63]^. Another class of cancer vaccines that can specifically target cancer stem cells (CSCs) is also one of the current research hotspots. Recent CSC vaccine research on murine melanoma stem cell (MSCs)-based vaccine was an IL-33 modified MSCs vaccine that could target the B16F10-CD44+CD133+ cells. Meanwhile, dendritic cells would mature after the vaccine is received and trigger CTL responses, together with the activation of the CD8+ T cells, which are the effector against tumors^[64]^.

Here, we have to mention a clinical trial of the mRNA vaccine in combination with pembrolizumab for treating tumors initiated by Moderna Inc. and Merck Sharp & Dohme LLC. Moderna developed mRNA-4157 (V940), an mRNA neoantigen tailored to the patient’s specific tumor DNA sequence to stimulate the body’s immune response. In a phase II trial of the combination, in patients with completely resected melanoma stage III and IV melanoma, the combination with mRNA-4157 reduced the risk of recurrence and death by 44% compared to Pembrolizumab alone. The 18-month recurrence-free survival (RFS) rate was 78.6% (combination) *vs.* 62.2% (pembrolizumab), with 18-month distant metastasis-free survival (DMFS) rates being 91.8% (combination) *vs.* 76.8% (pembrolizumab), respectively (NCT03897881)^[65]^. This promising outcome has now entered a phase 3 clinical trial (NCT05933577).

Thanks to the intersection of computer science and biotechnology, the invention of tumor vaccines has become more accessible. With big data and computer algorithms, it is possible to compute and design tumor vaccines formed by combinations of multiple antigens. Yazdani *et al.* developed a novel multiepitope peptide vaccine by combining computerized bioinformatics, molecular docking, and dynamic stimulation assessments that combine three immunogenic proteins, NYESO-1, gp-100, and MART-1, with multiple helper epitopes as adjuvants^[59]^. Although the actual effect of this vaccine against melanoma still needs to be proved by further experiments, it shows us the application and future trend of computers in vaccine design. Another research on melanoma vaccine described a strategy for cancer immunotherapy based on DNA nanodevices. The researchers designed and fabricated a DNA robotic nanostructure that effectively protects antigens and adjuvants *in vivo* and transports them to draining lymph nodes (dLNs). Within dLNs, the nanodevice can respond to and release antigens and adjuvants in an acidic environment, activate dendritic cells, and elicit a robust, tumor-specific CD8+ CTL response via the Toll-like receptor (TLR) pathway and antigenic peptide presentation^[58]^. Although this research is murine-based, it is worth mentioning that it has demonstrated the potential of DNA nanodevice vaccines in cancer immunotherapy.

### Bispecific antibodies

Bispecific antibodies (bsAbs) are artificially designed antibodies that can specifically bind to two antigens or epitopes simultaneously. BsAbs are a new frontier in tumor immunotherapy in the 21st century. These antibodies can directly connect T cells to tumor cells, enhancing the immune system’s attack on tumors. Compared with monospecific antibodies, bispecific antibodies have more robust specificity and targeting, which can reduce off-target toxicity. There have been a few bsAbs approved by the FDA today. Blinatumomab from Amgen is the first approved bispecific antibody for the treatment of adult relapsed or refractory precursor B-cell acute lymphoblastic leukemia (ALL)^[66]^; amivantamab from Johnson&Johnson is a bsAb for EGFR-positive metastatic NSCLC^[67]^; Tebentafusp is an FDA-approved bispecific fusion protein by Immunocore in 2022 for the treatment of HLA-A^*^02:01-positive patients with unresectable or metastatic uveal melanoma. In the open-label, phase 3 trial (NCT03070392) of Tebentafusp, the OS of patients who received Tebentafusp at 1 year was 73% and 36% of PFS at 6 months, both significantly higher than the control group^[68]^. In the latest clinical trial results (NCT03070392), the 3-year median OS was 21.6 months, significantly higher than the control group’s 16.9 months, demonstrating the long-term benefits of Tebentafusp for patients^[69]^.

### Combination immunotherapy

Combination immunotherapy, also known as cocktail immunotherapy or combination therapy, involves the simultaneous or sequential use of multiple immunotherapeutic agents to enhance the anti-cancer immune response. Combination immunotherapy aims to improve treatment efficacy, overcome resistance mechanisms, and achieve better outcomes for patients with various types of cancer, including melanoma. The rationale behind combination immunotherapy is to exploit different aspects of the immune response and the tumor microenvironment to achieve a more potent and sustained anti-cancer effect. A clinical trial (NCT03470922) demonstrated a combination immunotherapy of Relatlimab (LAG-3-blocking antibody)-nivolumab (PD-1-blocking antibody)^[70]^. The combination showed a 10.1-month PFS, while the progression-free survival of nivolumab alone was 4.6 months.

It is important to note that the specific combination therapies used in clinical practice may vary depending on the type and stage of cancer and the individual patient’s characteristics. Therefore, careful patient selection and close monitoring are essential to manage potential adverse events. However, to maximize the efficacy of combined therapy, it is more important to use the correct order of different therapeutic drugs, especially when both immunotherapy and targeted therapy can significantly improve the patient’s OS. The DREAMseq stage III trial answers this question (NCT02224781). Patients with advanced BRAFV600-mutant metastatic melanoma were involved in this 2-step clinical trial to compare the effect of different dosing sequences on treatment efficacy^[71]^. Out of a total of 265 patients, only 73 patients went onto the step 2 trial. Of these, 27 patients continued to receive Dabrafenib (BRAF inhibitor) and Trametinib (MEK inhibitor) after first receiving Nivolumab and Ipilimumab. In comparison, 46 patients received Dabrafenib and Trametinib first, then Nivolumab and Ipilimumab. The 2-year OS of the former was 71.8% (95%CI, 62.5 to 79.1), while the 2-year OS of the latter was 51.5% (95%CI, 41.7 to 60.4). The DREAMseq trial confirmed that preferred nivolumab/ipilimumab followed by BRAF and MEK inhibitor therapy has better control of melanoma, providing a practical clinical treatment reference.

In therapy widely used with anti-PD-1 monoclonal antibodies, especially for patients with stage III/IV melanoma, the combined treatment of Nivolumab and Ipilimumab has significantly increased patient response rates. In a randomized clinical trial of patients with untreated stage III/IV melanoma, patients were assigned to receive initial treatment with nivolumab plus ipilimumab, followed by continued treatment with nivolumab and ipilimumab switching to placebo, or treatment with ipilimumab plus nivolumab placebo. At least five years of follow-up, the 5-year OS was 52% in the nivolumab plus ipilimumab combination group compared with the ipilimumab group, which was significantly higher than the 44% treated with nivolumab alone and the 26% treated with ipilimumab alone. These results suggest that the combination of nivolumab and ipilimumab demonstrated good long-term survival efficacy in this clinical trial^[71,72]^. For melanoma patients who have progressed to lymph node metastases or have both primary and metastatic lesions, Nivolumab plus ipilimumab may be an effective neoadjuvant treatment strategy to help control lesions and metastases, thereby providing patients with better surgical conditions. However, it is essential to note that this treatment strategy is not suitable for all patients and should be individualized on a case-by-case basis (NCT02437279, NCT02977052)^[73]^. A subsequent clinical trial, CheckMate-915, evaluated using Nivolumab combined with ipilimumab versus nivolumab alone as adjuvant therapy in patients with stage IIIB-D or IV melanoma resected. The results showed that in all randomized patient populations and patients with PD-L1 expression levels of less than 1%, nivolumab combined with ipilimumab did not improve RFS compared with nivolumab alone. In addition, the incidence of adverse events was higher in the combination treatment group^[74]^.

Furthermore, patients who received anti-CTLA-4 antibodies prior and continued to receive anti-PD-1 antibodies had more tumor mutation burdens than patients who did not experience anti-CTLA-4 therapy, which means an increase in the number of non-synonymous mutations in the genome of tumor cells that may lead to the production of neoantigens that activate the immune system’s attack on tumors. Among them, the most significant mutation is PIK3C2G missense. This may be because anti-CTLA-4 therapy (a) increases the immune recognition of tumor cells, leading to tumor cell death and the release of neoantigens, which may contain mutation sites; (b) alters the tumor microenvironment, increases the inflammatory state of the tumor, and promotes the accumulation of mutations; (c) affects the biological properties of tumor cells, for example, by altering DNA repair mechanisms or cell cycle regulation, increasing mutation rate^[75]^. A phase 2 clinical trial (Checkmate-204; NCT02320058) showed that the Nivolumab/Ipilimumab combination is also effective in melanoma patients with brain metastasis. The clinical results showed that the 6-month PFS rate was 64.2% intracranial and 75.9%, respectively. The 9-month PFS rates were 59.5% and 70.4%, respectively^[76]^. Clinical trials are ongoing to explore new combinations and optimize existing ones, aiming to provide more effective treatment options for patients with various types of cancer.

## MECHANISM OF RESISTANCE TO IMMUNOTHERAPY

**Figure 1:**
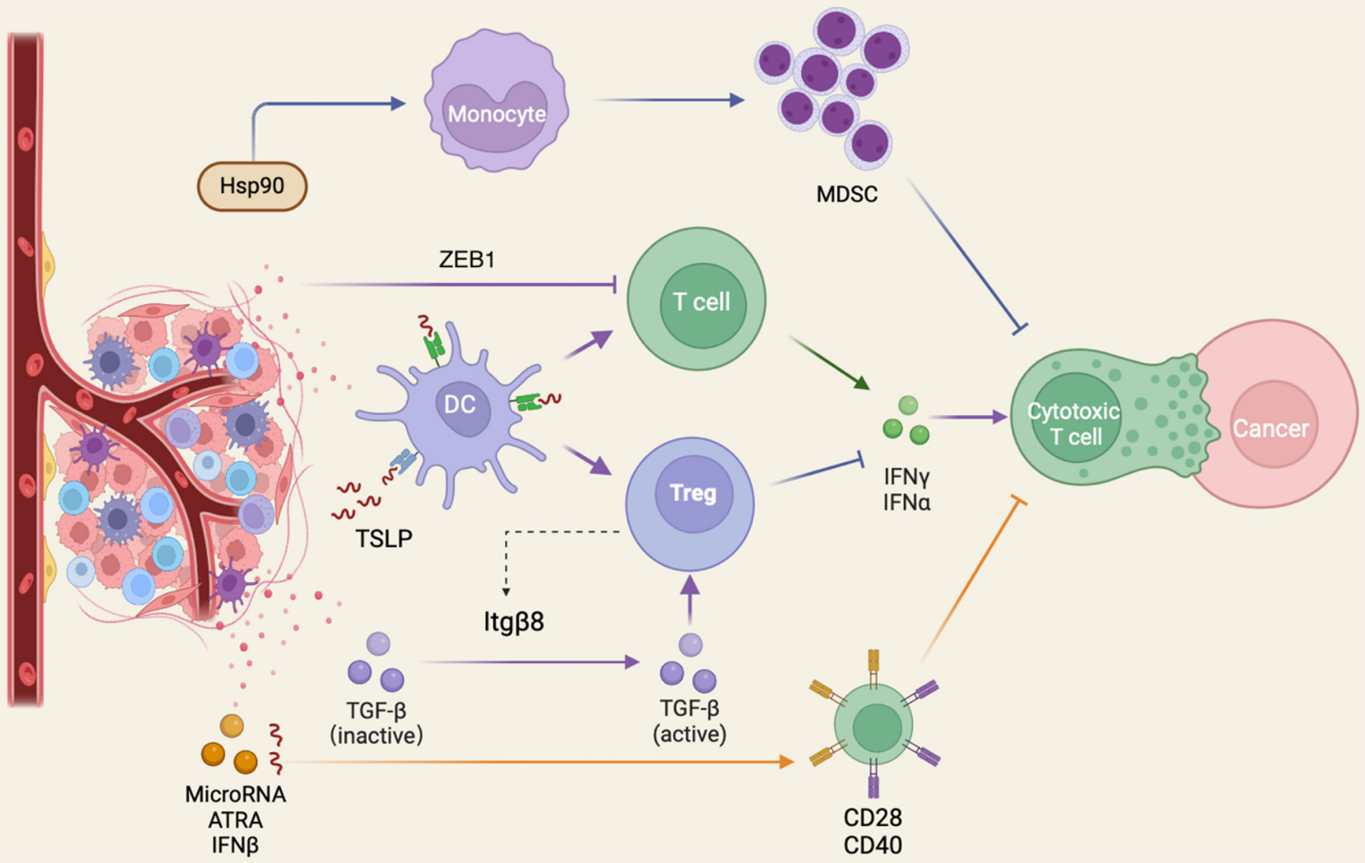
Mechanism of resistance to Immunotherapy. The tumor microenvironment of melanoma secretes various cytokines, chemokines, growth factors, and microRNAs that inhibit the function of immune cells or promote the recruitment of immunosuppressive cells. MDSC: Myeloid-derived suppressor cells; TSLP: thymic stromal lymphopoietin; Treg: regulatory T cell; IFN: interferon; ATRA: all-trans retinoic acid. The figure was created with Biorender (http://www.biorender.com).

While immunotherapy has significantly improved the treatment outcomes for many melanoma patients, not all patients respond to immunotherapy, and some may develop resistance over time. The underlying mechanisms for such disparities in treatment outcomes are intricate and multifaceted. Understanding these mechanisms is crucial for developing strategies to overcome immunotherapy resistance in melanoma and other cancers. Based on this knowledge of resistance, scientists are actively studying these processes to identify new targets and combination therapies that can enhance the effectiveness of immunotherapy and improve patient outcomes. A comprehensive depiction of the resistance mechanism of cancer immunotherapy can be found in Figure 1, offering readers a visual insight into these complex processes.

### Tumor microenvironment changes

The tumor microenvironment (TME) is a complex and dynamic ecosystem surrounding cancer cells. Within this environment, various cell types, including immune cells, fibroblasts, and blood vessels, interact with the tumor^[77]^. Melanoma cells can exploit this microenvironment to evade immune surveillance by secreting various soluble factors that create an immunosuppressive microenvironment. These factors may include cytokines, chemokines, and growth factors, as well as microRNAs that inhibit the function of immune cells or promote the recruitment of immunosuppressive cells. Additionally, the tumor microenvironment may be enriched with factors that induce the differentiation of immune cells into regulatory or tolerogenic phenotypes, further dampening the antitumor immune response. They may release chemical signals that attract immunosuppressive cells such as regulatory T cells (Tregs) and myeloid-derived suppressor cells (MDSCs). These suppressive immune cells create an immunosuppressive shield around the tumor, preventing the effective activation and functioning of antitumor immune cells^[78-80]^.

Melanoma induces keratinocytes to generate cytokine thymic stromal lymphopoietin (TSLP), transmits signals through the TSLP receptor of DC, promotes the expression of GATA3+Treg, and inhibits the activity of IFN-γ and the proliferation of CD8+ T cells. In lung squamous cell carcinoma (LUSC), it has also been found that the tumor produces immunosuppression through high expression of anti-inflammatory factors, such as TGFβ and CCL18^[81-84]^. Studies have found that Treg can cooperate with cancer cells to suppress immune activity. Inactive TGFβ secreted by cancer cells into the TME is activated by the β8 chain of αvβ8 integrin (Itgβ8) secreted by Treg^[85]^. Activated TGFβ promotes Treg development and suppresses effector T cell cytotoxicity in mammals. Arkhypov *et al.* demonstrated that soluble heat-shock protein 90α (HSP90α) prompts monocytes to become MDSC via TLR4 signaling and PD-L1 expression, potentially offering a target for countering immunosuppression in melanoma and improving immune-based therapies^[86]^. It was observed in melanoma patients that ATRA and IFN-β induce the expression of CD38 in the tumor microenvironment after treatment with anti-PD-1 antibodies, which inhibit the function of CD8+ T cells through adenosine receptor signaling, which may lead to resistance to anti-PD-1 therapy^[87]^.

MicroRNA (miRNA) plays a significant role in tumors by being secreted into the tumor microenvironment (TME) via exosomes to regulate the immune environment and facilitate immune escape. In one study, two miRNAs, hsa-miR-498 and hsa-miR-3187-3p, are found in melanoma exosomes and dampen immune responses by altering CD8+ T cell behavior. These miRNAs affect CD45 expression, a key component in T cell signaling, leading to reduced TNFα secretion. The study also identified 206 distinct miRNAs in melanoma exosomes and proposed a shared motif guiding miRNA sorting^[88]^.

Metabolic changes in tumor cells and T cells also play an important role in immunotherapy resistance in melanoma. Tumor cells adapt to the microenvironment and evade immune surveillance by regulating their own metabolism. For example, tumor cells can undergo aerobic glycolysis, similar to the metabolic demands of effector T cells, leading to nutrient competition between the two^[89]^. In addition, metabolites such as lactate secreted by tumor cells can alter the TME and inhibit the metabolism and cytotoxicity of T cells, thereby affecting the effect of immunotherapy. The function of T cells in the tumor microenvironment is also affected by metabolic reprogramming. Studies have shown that the metabolic state of the tumor microenvironment can inhibit the metabolism and cytotoxicity of CD8+ T cells. For example, lactate secreted by tumor cells alters the metabolism of CD8+ T cells by inhibiting pyruvate carboxylase (PC) activity, weakening their cytolytic activity. In addition, succinic acid secretion in the tumor microenvironment and autocrine activation of succinate receptor 1 (SUCNR1) play an important role in maintaining the cytotoxicity of CD8+ T cells^[90]^.

In addition to secreting various cytokines to regulate the TME, melanoma cells can upregulate immune checkpoint molecules, such as PD-L1, as a protective mechanism^[91]^. While the PD-1/PD-L1 pathway has been a primary target for immunotherapy, other immune checkpoints can be upregulated in melanoma cells. For example, T cell immunoglobulin and mucin domain 3 (TIM-3) and LAG-3 are additional inhibitory receptors expressed on T cells^[92-94]^. TIM-3 is a negative costimulatory molecule widely expressed in various immune cells. It mediates TIM-3+ T cell dysfunction and T cell exhaustion by binding to the ligand Gal-9, thereby inhibiting the antitumor immune response of T cells^[95]^. In the tumor microenvironment, especially in tumor-infiltrating T cells, co-expression of TIM-3 and LAG-3 correlates with the exhaustion status of T cells. T-cell function can be further suppressed when these receptors interact with their respective ligands on tumor cells. This redundancy in immune checkpoint pathways provides an alternative route for melanoma cells to evade immune destruction. As mentioned in the ICI section above, LAG-3 is a third-generation checkpoint, and outstanding achievements have been made in the research and development of its inhibitors. Hopefully, TIM-3 combined with LAG-3 blockade would also be an active treatment option.

### Loss of immunity tracking and response

Immunotherapy relies on the ability of the immune system to recognize specific antigens present in cancer cells. These antigens act as flags, signaling the immune system to attack the tumor.

#### Antigen loss

Melanoma cells can evolve and undergo genetic changes that result in the loss or downregulation of these antigenic markers^[96-98]^. Consequently, the immune system loses its targets, and the tumor becomes “invisible” to the immune cells, rendering immunotherapy less effective. This phenomenon, known as antigen loss, is a significant hurdle in generating sustained antitumor immune responses^[99]^.

#### Impaired antigen presentation

Antigen-presenting cells (APCs), particularly dendritic cells, play a crucial role in activating T cells by presenting tumor antigens to them^[100,101]^. This process can be disrupted in melanoma, leading to a reduced immune response. Melanoma cells may produce factors that inhibit dendritic cell maturation or migration to the lymph nodes, where they interact with T cells. Consequently, the presentation of tumor antigens to T cells is impaired, diminishing the chances of initiating a robust antitumor immune reaction^[102]^.

#### T-cell exhaustion

T cells are critical players in the immune response against cancer. T cells become activated upon recognizing tumor antigens and attack the cancer cells. However, the prolonged exposure of T cells to the tumor microenvironment can lead to exhaustion, where they lose their effector functions and become less responsive to antigens^[103]^. This T cell exhaustion is driven by persistent antigen stimulation and the engagement of inhibitory receptors, such as PD-1 and CTLA-4. The interaction between PD-L1 on tumor cells and its receptor PD-1 on T cells leads to T cell exhaustion and immune tolerance, further aiding the tumor in evading immune attacks^[104]^. As a result, the exhausted T cells fail to mount a potent and sustained immune attack against the melanoma cells.

#### Role of tumor-infiltration lymphocytes

TILs are immune cells that have successfully penetrated the tumor microenvironment. High levels of TILs have been associated with better responses to immunotherapy. However, not all melanomas have significant TIL infiltration, which can limit the effectiveness of immunotherapy. Strategies to enhance TIL recruitment and activation are areas of ongoing research. For instance, high expression of ZEB1 is associated with a reduction in CD8+ T cell infiltration in human melanoma samples. By implementing an increase or decrease in ZEB1 function in a mouse model, the study found that ZEB1 regulates tumor growth by controlling the recruitment of CD8+ T cells in tumors. In addition, ZEB1 directly inhibits the secretion of chemokines that attract T cells, including CXCL10. These findings suggest that ZEB1 is an essential factor in regulating immune escape in melanoma, and strategies targeting ZEB1 may help improve the efficacy of immunotherapy^[105]^.

While the immune system is adept at tracking and responding to melanoma, the tumor’s adaptive mechanisms can hinder this process. Understanding and countering these evasive tactics will be paramount in refining and enhancing immunotherapeutic approaches for melanoma.

### Heterogeneity-based immune-escape

Melanoma is a genetically heterogeneous disease, and tumors can harbor various subpopulations with distinct genetic profiles. Some subpopulations may be less susceptible to the selected immunotherapy due to the lack of targetable antigens or alterations in immunoregulatory pathways^[106]^. The primary mutations discussed in melanoma research include BRAF, PTEN, and NRAS mutations.

#### BRAF mutations

Clinically, the most prevalent mutation is the BRAF mutation, accounting for 50% of all mutations, and BRAFV600E accounts for 90% of BRAF mutations. BRAF mutations induce cell proliferation and migration by activating the MAPK signaling pathway. BRAF inhibitors have been widely used as targeted drugs, such as dabrafenib, vemurafenib, and encorafenib. However, the tumor heterogeneity of melanoma could easily lead to off-target effects of these drugs. Some common drug combinations are used to deal with tumor heterogeneity, such as BRAF/MEK inhibitors. Tian *et al.* also combined a BRAF/MEK inhibitor with an anti-PD-1 inhibitor in treating colorectal cancer (NCT03668431)^[107]^. Clinical trials combine various tumor drugs to find a treatment combination with higher efficiency. However, discovering the immune escape mechanism caused by different mutations is the fundamental way to solve resistant problems.

#### PTEN mutations

*PTEN* mutations belonging to the PI3K pathway can trigger immune evasion in melanoma. *PTEN* mutation not only reduces the cytotoxicity of T cells but also indirectly affects the functions of other immune cells, such as reducing the immune activity of B cells. In addition, some melanomas have low microphthalmia-associated transcription factor (MITF) expression. In the melanomas with simultaneous deletion of MITF and PTEN, there is a more obvious reduction of T cell recruitment. In addition, MITF^low^/PTEN^neg^ also reduces the expression of Major Histocompatibility Complex (MHC)-related genes, thereby causing resistance to immune checkpoint inhibitors^[108]^.

#### Loss of MHC antigen

MHC antigens play a vital role in the immune system, and they are critical factors in immune cell recognition and clearance of tumor cells. MHC molecules, especially MHC class I and II molecules, play a central role in the antigen presentation process. They can present antigenic fragments inside tumor cells to T cells, thereby activating T cell-mediated immune responses. The absence or decreased expression level of MHC molecules can weaken the recognition and attack of tumor cells by T cells, leading to immune escape. Melanoma cells can reduce the expression of MHC molecules through a variety of mechanisms. For example, they may silence *MHC* gene expression through epigenetic regulation, such as DNA methylation. In addition, specific cytokines in the tumor microenvironment may also affect the expression and function of MHC molecules. Deletion or downregulation of MHC antigens limits immunotherapy’s effectiveness, as immune checkpoint inhibitors (e.g., PD-1/PD-L1 inhibitors) depend on T cell recognition of tumor antigens. If MHC molecule expression is insufficient, T cells may not be able to recognize tumor cells effectively. They thus cannot be activated or maintained in an activated state, leading to resistance to immunotherapy^[16]^. The relationship between the loss of MHC-I antigen presentation mechanism and immune evasion could also be demonstrated in studies of melanoma resistance to MAPK inhibitor therapy. The study found that during the drug resistance phase of treatment, the expression of MHC-I molecules on the surface of tumor cells decreased, resulting in a decrease in T cell infiltration, thereby weakening the antitumor immune response. These results suggest that MHC-I molecules play a vital role in the tumor immune microenvironment and that activating signaling pathways such as MAPK, PI3K-mTOR, and Wnt is associated with the downregulation of MHC-I molecular expression. These findings provide a new therapeutic strategy to overcome resistance to MAPK inhibitors and immune checkpoint blockade therapies in melanoma^[109]^.

#### Other genetic factors

Besides a few well-studied melanoma mutations, many genetic mutations or deletions of different functions are associated with immunotherapy resistance. Previous studies have demonstrated that ipilimumab promotes IFN-γ production and can promote T cell proliferation in a tumor environment, helping immunotherapy. However, Gao *et al.* analyzed the clinical data of patients who did not respond to ipilimumab and found that gene defects in the IFN-γ pathway lead to melanoma’s response to immune checkpoint inhibitors, especially anti-CTLA-4 Antibody insensitivity^[110]^. Moreover, as the tumor grows and responds to treatment, it can accumulate new genetic mutations, including those involved in immune evasion. This genetic diversity within the tumor can pose challenges for treatment, as different subclones may respond differently to immunotherapy^[111]^. The highly adaptive nature of tumors results in some cancer cells with specific genetic mutations or resistance-conferring changes surviving and proliferating when the immune system targets and eliminates vulnerable tumor cells^[112]^. Over time, these drug-resistant cells can dominate the tumor mass, leading to treatment failure. The ability of tumors to “escape” the immune response is an ongoing challenge in cancer immunotherapy.

## MOLECULAR BIOMARKERS OF IMMUNOTHERAPY

Immunotherapy has emerged as a game-changer in cancer treatment, offering the promise of durable responses and improved survival for patients with various malignancies. However, the efficacy of immunotherapy is variable, and not all patients benefit equally from these novel treatments. They identified reliable predictive and prognostic biomarkers to determine which patients are most likely to respond to immunotherapy and tailor treatment strategies accordingly^[113,114]^. This section will explore the current state of predictive and prognostic biomarkers for immunotherapy and their potential role in guiding personalized cancer treatment [Table 1].

**Table 1:** Summary of molecular biomarkers

	**Biomarkers**	**Biological context**	**Correlation** **with response**	**Ref.**
**Predictive** **biomarkers**	PD-L1	A ligand expressed on tumor cells that binds to the PD-1 receptor on T cells	+	[116-120]
	TMB	Number of somatic mutations per coding area of a tumor genome	+	[121-123]
	MSI	Accumulation of errors in repetitive DNA sequences	+	[124-126]
	TLS	Immune cell aggregates present in non-lymphoid tissues	+	[127-129]
	TA-HEC	High endothelial venules where immune cells enter the tumor site	+	[130,131]
	Immune cell subsets	TREM2 macrophage subsets, γδ T cell subsets, B cell subsets, *etc.*	-	[132]
**Prognostic** **biomarkers**	TILs	Immune cells that have migrated into the tumor microenvironment	+	[133-135]
	Immune-related genes	Gene sets obtained through data analysis that could influence immune infiltration scores	+	[136]
	Gut microbiome	Gut microbiome influences immunotherapy’s efficacy	+	[137-139]
	FAM	Fatty acid synthesis and its transportation	-	[140-142]
	HHLA2	Immune checkpoint of the B7 family	+	[143-145]
**Circulating biomarkers**	Serum LDH	Prognostic/predictive	-	[146-148]
	The NLR	Prognostic	-	[149,150]
	CRP	Prognostic	-	[151,152]
	Diversity and clonality of TCRs	Prognostic/predictive	+	[153,154]
	Circulating EVs	Predictive	-	[155-158]
	Blood cell composition	Predictive	+/-	[159]

TMB: Tumor mutational burden; MSI: microsatellite instability; TLS: tertiary lymphoid structures; TA-HEC: tumor-associated high endothelial cells; TILs: tumor-infiltrating lymphocytes; FAM: fatty acid metabolism; HHLA2: HERV-HLTR-associating 2; LDH: lactate dehydrogenase; NLR: neutrophil-to-lymphocyte ratio; CRP: C-reactive protein; TCRs: T cell receptors; EVs: extracellular vesicles.

### Predictive biomarkers

Predictive biomarkers are biomolecular or genetic characteristics that help predict a patient’s response to a specific treatment. In the context of immunotherapy, these biomarkers can assist in identifying patients who are more likely to benefit from ICIs or other immunotherapeutic agents. Predictive biomarkers can spare patients from unnecessary treatments and associated toxicities while ensuring that those most likely to respond receive timely and targeted therapies^[115]^.

One of the most extensively studied predictive biomarkers for immunotherapy is PD-L1 expression on tumor cells. PD-L1 is a ligand expressed on tumor cells that binds to the PD-1 receptor on T cells, suppressing their immune response^[116]^. High levels of PD-L1 expression have been associated with improved responses to anti-PD-1/PD-L1 therapy in multiple cancer types, including melanoma, non-small cell lung cancer, urothelial carcinoma, triple-negative breast cancer, gastric adenocarcinoma, and ovarian cancer^[117,118]^. Based on determining PD-L1 expression levels, physicians can better decide which patients may benefit from immune checkpoint inhibitor therapy. For example, the approval of certain drugs is based on specific thresholds for PD-L1 expression levels, such as the PD-L1 Tumor Proportion Score (TPS) of ≥ 1% when using pembrolizumab in patients with non-small cell lung cancer. Changes in the expression level of PD-L1 in the tumor treatment stage can also help with precision therapy. For example, in a mouse model of melanoma MAPK-targeted treatment, it was found that the loss of ITCH expression in tumor cells can lead to upregulation of PD-L1 expression, decreased infiltration of CD8+ T cells within tumors, and increased number of regulatory CD4+ T cells and type 2 tumor-associated macrophages, thereby promoting tumor escape immunosuppression and generating treatment resistance, while high expression of ITCH can inhibit the upregulation of PD-L1 during treatment and maintain CD8+ effector T in tumors Proliferation and activity of cells, so activating the E3 ubiquitin ligase activity of ITCH can be used as a combination therapy with MAPK-targeted therapy^[119]^. However, the clinical utility of PD-L1 as a sole predictive biomarker is limited by variable expression levels across tumor types and heterogeneity within individual tumors^[120]^. PD-L1 expression may not be sufficient as a potent biomarker in some rare tumor subtypes. For example, some tumors may achieve immune evasion through other pathways, making the association between PD-L1 expression and treatment response not obvious.

Tumor mutational burden (TMB), the number of somatic mutations per coding area of a tumor genome, has emerged as a potential predictor of immunotherapy response^[121]^. Cancers with high TMB tend to produce more neoantigens, which the immune system recognizes as foreign, increasing the likelihood of an antitumor immune response. Several studies have shown that patients with high TMB exhibit improved responses to immunotherapy, irrespective of the tumor type^[122,123]^.

Microsatellite instability (MSI) is another promising biomarker associated with immunotherapy response. MSI results from deficient DNA mismatch repair, leading to an accumulation of errors in repetitive DNA sequences^[124,125]^. MSI-high tumors are more responsive to immunotherapy, as these tumors generate a higher load of neoantigens, enhancing the immune system’s ability to recognize and target cancer cells^[126]^.

Tertiary lymphoid structures (TLS) are immune cell aggregates in non-lymphoid tissues, usually in an inflammatory environment, and have been found in various cancers, including melanoma. TLS have CD20+ B cells wrapped by CD4+ T cells^[127,128]^. The study found that TLS-high tumors are more sensitive to anti-CTLA-4 antibodies, indicating a higher overall survival rate after receiving anti-CTLA-4 antibodies^[129]^.

After cancer patients receive ICI therapy, immune cells enter the tumor site through tumor-associated high endothelial venules (TA-HEVs), and their endothelial cells (TA-HEC) are associated with T-cell infiltration^[130]^. The biopsy analysis of 93 melanoma patients proved that the higher the frequency and maturity of TA-HEC, the better the survival rate of patients receiving the combination of anti-PD-1 antibody and anti-CTLA-4 antibody^[131]^.

In addition to finding a biomarker positively correlated with ICI efficacy, finding a biomarker that can predict ICI non-response will provide another basis for prediction. Analysis of a single-cell sequencing data set of melanoma samples found that patients who did not respond to ICI therapy had overexpressed TREM2 macrophage subsets and γδ T cell subsets and had fewer B cell subsets^[132]^.

### Prognostic biomarkers: beyond response prediction

While predictive biomarkers focus on identifying patients who are likely to respond to immunotherapy, prognostic biomarkers provide information about the patient’s overall disease outcome, regardless of treatment response. These biomarkers can aid in understanding the patient’s prognosis and inform treatment decisions beyond immunotherapy^[113,114]^.

One of the well-established prognostic biomarkers in cancer is TILs. TILs are immune cells that have migrated into the tumor microenvironment and play a crucial role in antitumor immune responses^[133]^. High levels of TILs have been associated with better prognosis and improved responses to immunotherapy in various cancer types, including melanoma and breast cancer^[134,135]^.

In addition to immune cells as prognostic markers, some studies have also screened a series of immune-related genes to obtain a gene set that can effectively respond to immunotherapy. Huang *et al.* screened 63 immune genes related to melanoma prognosis, sorted out a gene set with eight immune genes (*PSME1*, *CDC42*, *CMTM6*, *HLA-DQB1*, *HLA-C*, *CXCR6*, *CD8B*, *TNFSF13*) as melanoma prognostic markers, and verified their good accuracy as prognostic markers by using TCGA cohort^[136]^.

The gut microbiome has emerged as a potential prognostic biomarker for immunotherapy response^[137]^. Studies have shown that the gut microbiome’s composition can influence immunotherapy’s efficacy, as certain bacterial species can modulate the systemic immune response^[138]^. Understanding the role of the gut microbiome in immunotherapy outcomes could open new avenues for optimizing treatment responses. A recent metagenomic sequencing study of stool samples from 106 of 120 patients who participated in a clinical trial of the combination of ipilimumab and nivolumab found that gut microbial abundance could improve patient response to ICI. The study also points to a specific strain of Faecalibacterium associated with a positive reaction to combined ICI therapy in cancer patients^[139]^. This study highlights the importance of gut microbiome strain level analysis for understanding and predicting the efficacy of ICI therapy in the gut microbiome. It provides new insights for the development of microbiome-based cancer treatment strategies.

Fatty acid metabolism (FAM) is an integral part of the human body’s metabolic pathway and is cells’ main energy-generating metabolic pathway. Dysregulation of fatty acid metabolism can be found in various tumors^[140]^. A study screened nine FAM-related lncRNAs from the TCGA database as prognostic biomarkers for female cervical cancer^[141]^. Xu *et al.* also screened a group of FAM molecular subtypes through the TCGA database, in which six significant genes (*ACSL5*, *ALOX5AP*, *CD1D*, *CD74*, *IL4I1*, and *TBXAS1*) can act as biomarkers for melanoma prognosis^[142]^. Tumors with higher levels of expression have a higher sensitivity to chemotherapy. At the same time, high gene expression predicts tumors’ immune evasion ability and is highly correlated with PD-1 signaling pathways, T cell antigen presentation, and immune checkpoints. Therefore, the high expression of these genes also indicates a poor response to ICI.

HERV-HLTR-associating 2 (HHLA2) is an immune checkpoint of the B7 family^[143]^. HHLA2 can be a prognostic biomarker in various cancers, such as ovarian and pan-cancer (all cancers)^[144]^. Huang *et al.* examined the relationship between HHLA2 expression and the prognosis of melanoma patients^[145]^. They found a positive correlation between HHLA2 and CD8+ levels in tissues from 81 patients with advanced unresectable melanoma. Melanoma with high expression of HHLA2 predicts a better prognosis.

### Circulating biomarkers

Compared with biomarkers that need to be obtained by complex testing methods such as immunohistochemistry, H&E staining, and next-generation sequencing, circulating biomarkers are a series of non-invasive biomarkers, usually obtained through peripheral blood or body fluid testing. The information acquisition is relatively easy and more conducive to early detection and real-time monitoring of treatment prognosis.

Serum lactate dehydrogenase (LDH) is one of the tumor prognostic biomarkers, and elevated LDH levels predict poor prognosis for tumor immunotherapy. LDH is also considered an effective predictive and prognostic biomarker in cases of melanoma^[146,147]^. However, the significance of LDH changes under different treatments still needs to be accumulated through research data. In a retrospective study of patients with malignant melanoma in multiple melanoma centers around the world, patients with BRAFV600 mutations with elevated LDH levels who received anti-PD-1 antibody and anti-CTLA-4 antibody had longer PFS and OS than patients treated with first-line targeted therapy or a single ICI. Still, in BRAF wt patients with high levels of LDH, combination therapy is not significantly superior to ICI alone, which also indicates some limitations of LDH as a biomarker for melanoma^[148]^.

The neutrophil-to-lymphocyte ratio (NLR) is an essential parameter in blood counts that reflects the inflammatory status and immune surveillance balance. In a variety of solid tumor patients, an elevated NLR is often associated with a worse prognosis. During melanoma immunotherapy, changes in NLR may indicate treatment response and patient survival, and a decrease in NLR may be associated with treatment response and longer PFS^[149]^. The rationale behind NLR as a biomarker has also been discovered in the latest retrospective study. Transcriptome analysis showed that genes associated with elevated NLR, such as CD39, CCNA1, LDHA, and IL18R1, played a role in immunosuppression and inflammation, which could be the possible reasons for the poor prognosis of high NLR^[150]^.

C-reactive protein (CRP) is an acute-phase protein usually elevated in response to inflammation or infection. In melanoma immunotherapy, elevated CRP levels may reflect the body’s immune response to the tumor. Some studies have pointed out that CRP levels are related to the efficacy of immunotherapy and patient survival and that patients with higher CRP levels may have poor treatment outcomes^[151,152]^.

The diversity and clonality of T cell receptors (TCRs) are important indicators for assessing T cell immune responses. In melanoma immunotherapy, TCR diversity and clonality analysis can help to understand tumor-specific T cell activity and the efficacy of immunotherapy. Studies have shown that increased TCR diversity after treatment may be associated with better treatment response and survival^[153]^. In addition, further studies have shown that TCR repertoire metrics in tumor-infiltrating T lymphocytes (TIL/Tc) diversity and clonality have prognostic and predictive effects on OS and anti-PD-1 antibodies, respectively. High TIL/Tc diversity enables the identification of patients who can control tumors without PD1 inhibitors. Thus, further research on this approach will provide crucial information on the choice of treatment options for melanoma^[154]^.

Circulating extracellular vesicles (EVs) are vesicles composed of lipid bilayers, which can contain a variety of biomolecules, such as proteins, lipids, receptors, *etc.* Apoptotic bodies are often considered larger vesicles originating from the cell membrane’s budding and containing cellular debris. At the same time, exosomes are smaller EVs formed within the endosomes and released after fusion into the cell membrane^[155]^. EVs are believed to affect the tumor microenvironment and the immune system’s response against tumors^[156]^. The surface of the EV membrane expresses PD-L1 and CTLA-4. Therefore, EVs will likely be used as predictive biomarkers^[157]^. Serratì *et al.* demonstrated that patients who do not respond to ICI therapy may have higher levels of PD-L1+EV and PD-1+EV (from tumor cells and immune cells, respectively), so they can serve as two predictive biomarkers for melanoma immunotherapy^[158]^.

Additionally, blood cell composition in the circulating blood is also an essential non-invasive biomarker. Changes in the levels of CD8+ T Cells, CD4+ T Cells, Nature Killer cells, B cells, Myeloid cells, and circulating tumor cells in peripheral blood and the ratio between them have different degrees of predictive effect. Splendiani et al. provide a good summary of the ability of these cells to act as biomarkers^[159]^. The research and application of non-invasive circulating biomarkers bring significant advantages to cancer management, as they minimize risk and discomfort for patients by detecting blood or other bodily fluid samples. These biomarkers help enable early detection and diagnosis of cancer, facilitating patients to start treatment promptly, thereby improving survival. In addition, they can monitor the effect of treatment in real time, providing doctors with a basis for adjusting treatment plans and accurately assessing the patient’s prognosis. By identifying high-risk populations and guiding individualized treatment, circulating biomarkers improve the precision and effectiveness of treatment, help reduce healthcare costs, and improve patients’ quality of life. Its convenience and accessibility have further promoted the progress of dynamic cancer monitoring and research, opening up new possibilities for discovering new therapeutic targets and promoting the development of cancer treatment technologies.

While significant progress has been made in identifying predictive and prognostic biomarkers for immunotherapy, several challenges remain. The heterogeneity of tumors and their microenvironments and the dynamic nature of the immune response make biomarker discovery and validation complex^[160,161]^. In addition, the boundary between predictive and prognostic biomarkers is usually unclear, so when people discover a new biomarker, it is impossible to categorize it definitively as predictive or prognosis^[162]^. To address these challenges, multi-omic approaches, such as integrating genomics, transcriptomics, and proteomics data, are employed to identify comprehensive biomarker signatures^[163,164]^. Machine learning algorithms and artificial intelligence are also used to analyze large datasets and uncover novel associations between biomarkers and treatment responses^[165,166]^. Moreover, combining predictive and prognostic biomarkers may offer a more comprehensive assessment of patient outcomes and treatment responses^[167]^. Clinicians can tailor treatment strategies and optimize patient outcomes by integrating multiple biomarkers.

## PERSPECTIVES AND CONCLUSIONS

Immunotherapy has revolutionized the treatment landscape for melanoma, providing remarkable clinical benefits for many patients. Identifying predictive and prognostic biomarkers has further improved patient selection and treatment outcomes. However, challenges exist, such as resistance development and identifying the most effective treatment combinations. In this review, we conducted a multifaceted summary of studies related to melanoma immunotherapy in the past three years by searching the PubMed database. First, this review summarizes the six main immunotherapy strategies for melanoma at this stage and three major resistance mechanisms. The therapeutic effect of each immunotherapy varies, and to minimize the drug’s side effects and reduce the recurrence rate, it is clinically preferred to provide different combination therapy regimens according to each patient’s specific situation. Finding the proper treatment for each patient and giving precision treatment will become the mainstream of future research. Therefore, diverse and accurate biomarkers are essential. This review also summarizes the novel biomarkers published in the database in the past three years. These biomarkers are extensions of some common biomarkers and provide new ideas for subsequent biomarker research. The development of biomarkers will take immunotherapy for cancer to a new level. A variety of research methods are mentioned in this review, including basic research, prospective research, retrospective research, and clinical trials. Among them, it is inevitable that some retrospective studies may have problems with incomplete or incomplete records, and there may be potential bias in data collection, resulting in an inability to accurately reflect the actual situation, which leads to the questioning of the reliability of the study results. Recognizing these inherent limitations, this paper endeavors to mitigate them by carefully excluding low-quality studies during the article selection process. Furthermore, in certain clinical trials, the presence of samples that are either too small or too large, as well as the exclusion of prematurely terminated samples from the entire process, can undeniably introduce bias to the experimental results.

As melanoma immunotherapy evolves, several promising avenues deserve exploration to enhance treatment efficacy and patient outcomes. While immunotherapy has shown remarkable results, some patients still experience treatment resistance. Understanding the mechanisms underlying resistance to immunotherapy and developing strategies to overcome it will be vital in improving long-term responses and reducing relapse rates. In addition, immunotherapy is continuously advancing, and the discovery of novel immunotherapeutic approaches such as adoptive T-cell therapy, cancer vaccines, and oncolytic viruses should be further explored for their potential in melanoma treatment. Much of the melanoma immunotherapy research has focused on advanced or metastatic disease. Investigating the role of immunotherapy in earlier stages of melanoma may offer opportunities for better long-term disease control and potential cures. Continued monitoring of patients who have received immunotherapy is essential to gain insights into the durability of responses and possible long-term side effects. Understanding the long-term safety and efficacy profiles will aid in optimizing treatment strategies and managing patient expectations.

The future of immunotherapy for melanoma holds excellent promise. As research advances, we can expect more refined treatment strategies considering individual patient characteristics and tumor biology. Personalized medicine based on predictive biomarkers will likely become a standard approach, leading to increased response rates and improved survival. Moreover, novel therapeutic targets and combination therapies will emerge as we uncover the intricacies of the tumor microenvironment and the immune response. Collaborative efforts between clinicians, researchers, and industry stakeholders will be crucial in translating these discoveries into clinical applications. Ultimately, ongoing research and clinical trials will pave the way for continued progress in immunotherapy for melanoma, bringing us closer to improving patient outcomes and achieving long-lasting remissions of this devastating disease.

## DECLARATIONS

### Copyright

© The Author(s) 2024.
